# Formaldehyde in Solid Form

**Published:** 1898-01

**Authors:** William Rollins

**Affiliations:** Boston, Mass.


### FORMALDEHYDE IN SOLID FORM.

BY WILLIAM ROLLINS, BOSTON, MASS.

As you have considered formaldehyde of sufficient interest to dentists to publish a long article from one of the medical magazines, you may possibly find room for this short note on one of its dental applications. If we make a very strong aqueous solution of this gas, part of it slowly assumes the solid form and is precipitated. When this is dried and the pulp-chamber in a tooth filled with it, after a time it is all reconverted into a gas and thoroughly disinfects the whole tooth. If there is an absence at the root, by sealing in the solid formaldehyde with cement most of the gas escapes through the abscess, which soon yields to the treatment, which should be renewed every three days as long as required. An extended use has shown me the value of this treatment.

